# Sand fly and *Leishmania* spp. survey in Vojvodina (Serbia): first detection of *Leishmania infantum* DNA in sand flies and the first record of *Phlebotomus* (*Transphlebotomus*) *mascittii* Grassi, 1908

**DOI:** 10.1186/s13071-017-2386-z

**Published:** 2017-09-26

**Authors:** Slavica Vaselek, Nazli Ayhan, Gizem Oguz, Ozge Erisoz Kasap, Sara Savić, Trentina Di Muccio, Luigi Gradoni, Yusuf Ozbel, Bulent Alten, Dušan Petrić

**Affiliations:** 1 Faculty of Agriculture, Department of Phytomedicine and Plant Protection, Laboratory for Medical Entomology, University of Novi Sad, Novi Sad, Serbia; 2 UMR “Emergence des Pathologies Virales” (EPV: Aix-Marseille Univ - IRD 190 - Inserm 1207 – EHESP – IHU Mediterranee infection), Marseille, France; 3 Faculty of Science, Department of Biology, Ecology Division, VERG Laboratory, Hacettepe University, Beytepe, Ankara, Turkey; 4 Scientific Veterinary Institute “Novi Sad”, Novi Sad, Serbia; 5 Department of Infectious Diseases, Istituto Superiore di Sanità, Rome, Italy; 6 Faculty of Medicine, Ege University, Izmir, Turkey

**Keywords:** Sand fly, *Phlebotomus*, Leishmaniasis, *Leishmania infantum*, Serbia

## Abstract

**Background:**

Leishmaniasis in Serbia was an endemic disease, and is considered to be eradicated for more than 40 years. In the past decade sporadic cases of canine leishmaniasis started to emerge for the first time in Vojvodina Province (previously non-endemic region of Serbia). Reports of introduced, and later on autochthonous cases of leishmaniasis alerted the possibility of disease emergence. The aim of this study was to bridge more than a half a century wide gap in entomological surveillance of sand fly vectors in Vojvodina, as well as to verify the presence of the vector species that could support *Leishmania* spp. circulation.

**Results:**

During the period 2013–2015, a total of 136 sand flies were collected from 48 of 80 surveyed locations. Four sand fly species of the genus *Phlebotomus* were detected: *P. papatasi*, *P. perfiliewi*, *P. mascittii* and *P. neglectus.* Detection of *P. mascittii* represents the first record of this species for the sand fly fauna in Vojvodina and in Serbia. All female specimens (*n* = 80) were tested for *Leishmania* spp. DNA, and three blood-fed *P. papatasi* specimens were positive (4%). One positive DNA sample was successfully amplified by ITS1 nPCR. The RFLP analysis of the resulting 350 bp fragment showed a typical pattern of *L. infantum*, and the ITS1 partial sequence blasted in GenBank confirmed 100% identity with *L. infantum* and *L. donovani* complex sequences. This result represents the first record of both *Leishmania* spp. and *L. infantum* DNA from sand flies in Vojvodina, and in Serbia.

**Conclusions:**

Presence of autochthonous canine leishmaniasis cases, records of *Phlebotomus* (*Larroussius*) species proven vectors of *L. infantum* (*P. perfiliewi* and *P. neglectus*) and detection of *L. infantum* DNA from wild caught (non-competent) vectors, prove that *L. infantum* is present in Vojvodina and indicates a probable circulation in the region.

## Background

The leishmaniases are major vector-borne diseases caused by protozoan parasites belonging to the genus *Leishmania*. The parasites are transmitted to humans and other vertebrates by the bite of infected female sand flies (Psychodidae, Phlebotominae). Around one billion of people are at risk of infection while number of reported cases per year is estimated at 0.7–1.3 million for cutaneous leishmaniasis (CL) and 200,000–400,000 for visceral leishmaniasis (VL), causing over than 20,000 deaths annually [1].

In Europe, leishmaniasis is endemic in all southern countries, with ~700 (3950 if Turkey is included) autochthonous human cases reported every year [1]. The spread of *Leishmania infantum*, causative agent of zoonotic VL and CL in humans and domestic dogs (reservoir host) represents a major threat to Europe. Increasing dog and human travel, as well as the ongoing migrant crisis, pose a significant risk of *L. infantum* introduction into central Europe. Climate and land cover changes could also support northward dispersal of vectors, establishment of seasonal biting rates matching those of southern Europe, hence permitting autochthonous transmission [2].

In Serbia, leishmaniasis started to emerge after the Second World War. Due to the composition and abundance of sand fly species at that time, poor hygienic and health conditions in the post-war period, the disease rapidly assumed an epidemic character [3]. The first autochthonous case of VL was reported in 1945 [4]. From 1945 to 1955, leishmaniasis spread in epidemic waves from the southern parts of the country northwards, reaching its northernmost limit in central Serbia. After the last major epidemic (1953), the number of new cases started to diminish and, in the following years, the disease appeared only sporadically. The last case of VL was reported in 1968 [5], thereafter the disease was considered eradicated.

Sand fly research in Serbia was initiated in 1947, soon after first cases of autochthonous VL emerged, and was terminated in 1990. During this period, the presence of seven species of the genus *Phlebotomus* was recorded: *Phlebotomus papatasi*, *P. perfiliewi*, *P. tobbi*, *P. neglectus*, *P. simici*, *P. sergenti* and *P. balcanicus* [6]. The most intensive sand fly investigations were conducted during the VL epidemics, mainly in the infested areas. Following the disease spread, sand fly research was mainly focused on the south-east, east and central Serbia, leaving all other areas of country unexplored or partially explored. One of these less investigated and leishmaniasis-free region of Serbia includes Vojvodina Province, located in the north of the country.

Vojvodina was surveyed only briefly between 1949 and 1951. Research was conducted irregularly (through the years and seasons) and only in a small number of locations. Studies revealed the presence of only three species: *P. papatasi*, *P. perfiliewi* and *P. neglectus* [7]. The number of specimens caught was rather low, with several samples collected from various villages or from various houses in the same village. Low sand fly diversity and abundance, as well as the absence of human cases, resulted in the neglect of sand fly studies in this area for more than 60 years.

In the past decade, since 2006, cases of canine leishmaniasis have started to emerge for the first time in Vojvodina. Clinical symptoms and positive serological findings were first diagnosed in dogs that were imported or had travelled abroad to some of the Mediterranean countries with endemic leishmaniasis [8]. Subsequent findings (2010–2013) involved dogs that had never travelled from their home in Vojvodina [9]. This information suggested that both parasite and vector species are present in the region and implicated the possibility of autochthonous transmission.

The aim of this study was to bridge more than a half a century wide gap in entomological surveillance of sand fly vectors in Vojvodina, a very important transition region of Europe, as well as to verify the presence of the vector species that could support *Leishmania* spp. circulation.

## Methods

### Sand fly sampling

Cross-sectional entomological surveys were conducted between 2013 and 2015 in selected sample sites of the Vojvodina Province (North Serbia). A total of 80 villages were surveyed: 17 in 2013, 24 in 2014 and 39 in 2015. Surveys were conducted from the middle of May until the middle of September. Due to the restricted funding, sites sampled positive at first collection were not re-sampled, whereas negative locations were sampled again. Sampling locations were partly chosen according to the available data about sand fly presence obtained during previous investigations (1948–1951) and data regarding reported and/or suspected cases of canine leishmaniasis (2006–2013). The remaining locations are situated in areas without any data of sand fly and/or leishmaniasis presence.

Multiple sampling techniques were used to increase the number of specimens sampled, as low abundance was expected according to historical data. Indoor and outdoor populations of sand flies were collected using a miniature Centre for Disease Control (CDC) light traps (John W. Hock Company, model 512, Gainesville, Florida, U.S.A.), dry-ice baited traps without light (NS2 type), dry-ice baited traps with light, sticky papers and mouth aspirators. Suction traps were operating overnight, set at 16:00 h and collected at 08:00 h the next day. Traps were placed ~1.5 m above the ground inside (CDC) and outside (traps with dry ice) of the houses and animal shelters. Sticky traps (20 × 30 cm papers coated with commercial castor oil) were placed in holes surrounding walls of animal shelters and houses, and all other suitable sand fly resting places, for a period of four days. Indoor collections were performed during the daylight using mouth aspirators.

### Morphological identification of sand flies

All specimens collected were immediately transferred to 96% ethanol. Specimens were dissected; head and terminal segments of the abdomen were removed, cleared in Marc Andre solution and mounted in Berlese medium. The head and the tip of the abdomen were used for morphological identification, while the rest of the body was transferred to a separate tube with 70% ethanol and stored for DNA analyses. Morphological identification was based on characters of male genitalia, female spermathecae and pharyngeal armature [10, 11].

### Molecular identification of sand flies

In order to validate the morphological identification of sand fly specimens, sequence analysis of cytochrome *c* oxidase subunit 1 (*cox*1) mtDNA region was performed. PCR amplification was performed using the LCOI490/HCO2198 primer pairs according to the procedure of Folmer et al. [12]. The amplification products were electrophoresed trough 2% agarose gel and visualised under UV light. PCR products were purified using a QIAquick PCR Purification Kit (Qiagen, Hilden, Germany) and directly sequenced in both directions, using the same primers used for DNA amplification. Next-generation sequencing (NGS) was performed with BioRobot EZ1-XL Advanced (Qiagen). Sequences obtained were edited and aligned using BioEdit (7.0.9.0) [13] and compared with those available in GenBank using Neighbor Joining algorithm under the assumption of Kimura’s two parameter model in MEGA 6.06 [14].

### Detection of *Leishmania* spp.

Presence of leishmanial DNA was assayed individually in all females [15]. Small-subunit (SSU) ribosomal DNA was amplified by a sensitive nested (n) PCR technique using the Kinetoplastida and *Leishmania* genus-specific primers in the first and second PCR round, respectively [16]. Negative (no DNA) and positive (DNA from cultured *L. infantum* promastigotes) controls were used in all experiments. PCR products were electrophoresed through 1.5% agarose gel and visualized under UV light. Positive samples yielded a predicted nPCR product of 358 bp. *Leishmania*-positive DNA samples were then examined individually by internal transcribed spacer (ITS) 1 nPCR using LITSR/L.5.8S primers, and the amplified fragment analyzed by both restriction fragment length polymorphism (RFLP) and sequencing analysis for *Leishmania* species identification [17].

## Results

**Fig. 1:**
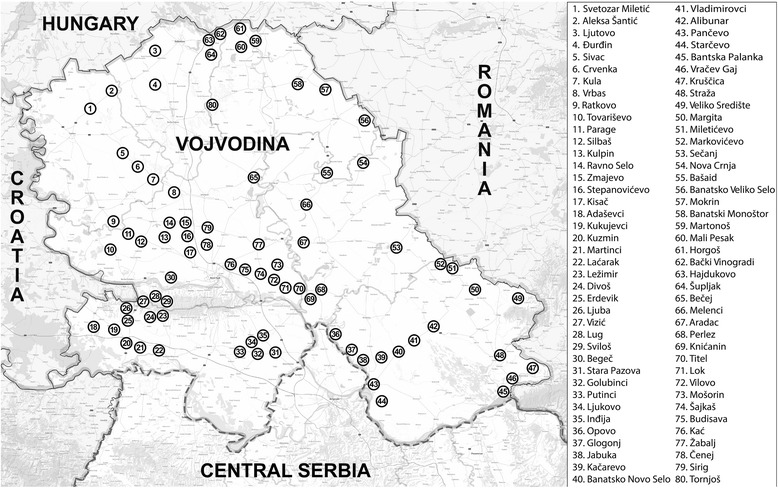
Sand fly collection localities in the study area

During 2013–2015, a total of 136 sand flies were collected from 48 of 80 locations (Fig. 1). Four sand fly species of the genus *Phlebotomus* were identified: *P. papatasi*, *P. perfiliewi*, *P. mascittii* and *P. neglectus* (Table 1). The majority of specimens (*n* = 130) belonged to *P. papatasi* (75 females, 54 males, 1 undetermined) (GenBank: KY848828), three females were identified as *P. perfiliewi* (GenBank: KY848829), two females as *P. mascittii* and one female as *P. neglectus* (GenBank: KY848830). *Phlebotomus papatasi* was the predominant species in Vojvodina, found in 55% of surveyed locations (91.67% of positive locations).

**Table 1:** Positive sand fly localities (coordinates, trapping dates, trap type, number of samples, species and gender)

Locality	Latitude	Longitude	No. of specimens	Date	Species and gender (male/female)	Positive trap type		
						CDC	CDC/CO_2_	CO_2_
Opovo^a^	45.0501220	20.4341260	8	21/07/2013	*P. papatasi* (1 m/5f)	×	–	–
				11/06/2014	*P. papatasi* (1 m/1f)	×	–	–
Inđija^a^	45.0353556	20.0790806	7	24/08/2013	*P. papatasi* (2 m/5f)	×	–	–
Golubinci	44.9836306	20.0715722	3	24/08/2013	*P. papatasi* (2 m/1f)	×	–	–
Putinci	44.9952167	19.9623000	10	24/08/2013	*P. papatasi* (2 m/8f)	×	–	–
Ljukovo	45.0295639	20.0227056	1	24/08/2013	*P. papatasi* (1 m)	×	–	–
Vladimirovci	45.0322167	20.8762667	20	21/07/2013	*P. papatasi* (7 m/13f)	×	–	–
Kačarevo	44.9623333	20.7128667	1	21/07/2013	*P. perfiliewi* (1f)	×	–	–
Bečej	45.6151970	20.0268460	2	24/07/2013	*P. papatasi* (1f)	–	–	×
				23/08/2015	*P. papatasi* (1 m)	–	–	×
Kać	45.3033710	19.9292900	4	25/07/2013	*P. papatasi* (1f)	×	–	–
				29/07/2013	*P. papatasi* (1 m)	×	–	–
				30/07/2013	*P. papatasi* (1 m/1f)	×	–	–
Begeč^a^	45.2380420	19.6228380	1	31/07/2013	*P. papatasi* (1 m)	×	–	–
Čenej	45.3692500	19.8042833	1	31/07/2013	*P. papatasi* (1f)	×	–	–
Stara Pazova	44.9839278	20.1797111	2	02/09/2015	*P. papatasi* (1 m/1f)	–	–	×
Budisava	45.2803167	19.9990833	1	22/07/2014	*P. papatasi* (1f)	×	–	–
Šajkaš	45.2664833	20.0925833	5	17/07/2014	*P. papatasi* (1 m/2f)	×	–	–
				22/07/2014	*P. papatasi* (2 m)	×	–	–
Žabalj	45.3755500	20.0761333	5	17/07/2014	*P. papatasi* (1 m/1f)	× (1f)	–	× (1 m)
				22/07/2014	*P. papatasi* (3 m)	× (2 m)	–	× (1 m)
Mošorin	45.2967333	20.1547333	2	26/07/2014	*P. papatasi* (1 m/1f)	–	–	×
Vilovo	45.2483500	20.1535500	1	26/07/2014	*P. papatasi* (1 m)	×	–	–
Perlez	45.2098500	20.3902333	2	26/07/2014	*P. papatasi* (2f)	×	–	–
Knićanin	45.1883167	20.3164167	2	26/07/2014	*P. papatasi* (1 m/1f)	×	–	–
Ljuba	45.1568333	19.3911167	1	19/08/2014	*P. papatasi* (1f)	×	–	–
Kuzmin	45.3040833	19.9285889	1	30/07/2014	*P. papatasi* (1f)	–	–	×
Vrbas	45.5809000	19.6322000	1	14/08/2014	*P. papatasi* (1f)	×	–	–
Kula	45.6178500	19.5127333	1	14/08/2014	*P. papatasi* (1f)	×	–	–
Lok	45.2183000	20.2113333	4	26/07/2014	*P. papatasi* (4f)	×	–	–
Lug	45.1876667	19.5429667	1	21/07/2015	*P. papatasi* (1 m)	–	–	×
Divoš	45.1115167	19.5095333	5	02/09/2015	*P. papatasi* (2f)	–	×	×
				21/07/2015	*P. papatasi* (2 m/1f)	–	×	–
Martinci	45.0132167	19.4452000	1	02/09/2015	*P. papatasi* (1 m)	–	×	–
Ležimir	45.1138333	19.5672500	3	02/09/2015	*P. papatasi* (1 m)	–	×	–
				18/06/2015	*P. mascittii* (1f)	–	×	–
				21/07/2015	*P. papatasi* (1f)	–	–	×
Bašaid	45.6356500	20.4080500	2	01/06/2015	*P. papatasi* (1 m)	–	×	–
				16/07/2015	*P. papatasi* (1f)	×	–	–
Nova Crnja	45.6698167	20.6086833	2	14/06/2015	*P. papatasi* (1 m/1f)	–	–	×
Banatsko Veliko Selo	45.8157167	20.6091500	1	16/07/2015	*P. papatasi* (1f)	–	×	–
Banatski Monoštor	45.9633000	20.2824167	1	16/07/2015	*P. perfiliewi* (1f)	–	×	–
Svetozar Miletić	45.8497833	19.1965667	1	20/08/2015	*P. neglectus* (1f)	–	×	–
Aradac	45.3768667	20.3025833	1	03/06/2015	*P. papatasi* (1 m)	–	–	×
Markovićevo	45.3249833	21.0331667	1	05/09/2015	*P. papatasi* (1f)	–	×	–
Miletićevo	45.3037167	21.0601500	1	22/08/2015	*P. papatasi* (1f)	–	–	×
Straža	44.9722500	21.3015000	1	16/06/2015	*P. papatasi* (1 m)	–	–	×
Vračev Gaj	44.8822000	21.3702167	2	22/08/2015	*P. papatasi* (1na)	–	×	–
				05/09/2015	*P. papatasi* (1 m)	–	×	–
Banatska Palanka	44.8463833	21.3344667	2	14/07/2015	*P. papatasi* (2 m)	–	–	×
Melenci	45.5282972	20.3037944	1	29/07/2015	*P. perfiliewi* (1f)	–	–	×
Parage	45.4154278	19.4045500	2	16/08/2015	*P. papatasi* (1f)	–	–	×
				01/08/2015	*P. mascittii* (1f)	–	–	×
Starčevo	45.4045000	19.7076333	1	28/06/2015	*P. papatasi* (1f)	–	–	×
Ravno selo	45.4525200	19.6197600	8	20/07/2015	*P. papatasi* (6 m/2f)	–	×	–
Ratkovo	45.4482900	19.3300200	3	19/07/2015	*P. papatasi* (3f)	–	× (1f)	× (2f)
Silbaš	45.3800600	19.5037600	1	19/07/2015	*P. papatasi* (1f)	×	–	–
Kulpin	45.3970900	19.5907200	2	20/07/2015	*P. papatasi* (2 m)	×	×	–
Zmajevo	45.4445400	19.6998900	1	20/07/2015	*P. papatasi* (1 m)	–	×	–
Tovariševo	45.3557600	19.3191400	6	19/07/2015	*P. papatasi* (2 m/4f)	–	×	–
Total: 48			Total: 136			80	31	25

*Abbreviations*: *f* female, *m* male, *na* unknown gender

^a^Localities where *Leishmania* spp. presence in sand flies was recorded

**Fig. 2:**
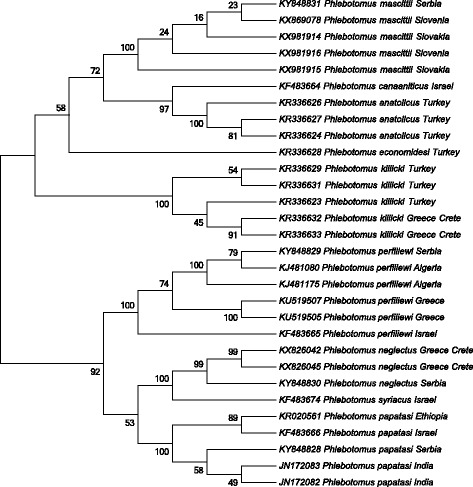
Sequence analysis based on the *cox*1 mitochondrial gene region in *Phlebotomus* species. Neighbor-joining tree based on the partial *cox*1 sequences used for molecular identification of the sand fly specimens

Presence of *P. mascittii*, a member of the *Phlebotomus* (*Transphlebotomus*) subgenus had not previously been reported from Vojvodina (or Serbia). During 2015, two female specimens of *P. mascittii* were collected in urban environment of Ležimir and Parage villages (geographical position given in Table 1). The first specimen was collected in Ležimir (18/06/2015) in a CDC/CO_2_ trap placed under a concrete roof that connects a brick storage shed and pig stain, creating a approximately 7 m tunnel like passage between the front (human) and back yard (animal dwelling). Mean monthly temperature for June in Ležimir were 18.6 °C at 07:00 h, 25.9 °C at 14:00 h and 19.1 °C at 21:00 h (source: national network of synoptic stations, station at Sremska Mitrovica, ~18 km from Ležimir). The second specimen was collected in Parage 01/08/2015 with a CO_2_ trap placed next to the brick wall of the house approximately 50 m from animals. Mean monthly temperature for August in Parage were 20.5 °C at 07:00 h, 30.6 °C at 14:00 h and 23.4 °C at 21:00 h (source: national network of synoptic stations, station at Rimski Šančevi, ~35 km from Parage). Both specimens were identified morphologically, and specimen identification was confirmed by sequence analysis of the *cox*1 mitochondrial gene region. Sequences obtained (GenBank: KY848831) were blasted against the database from GenBank and were identified as *P. mascittii* (Fig. 2). This report of *P. mascitii* represents the first record of this species in sand fly fauna of Vojvodina and in Serbia.

All female specimens (*n* = 80) were tested for *Leishmania* DNA; three blood-fed *P. papatasi* specimens were positive (4%). All positive females were collected in 2013 (3/37) from different settlements (Golubinci, Inđija and Opovo), thus resulting in 8.1% infection prevalence in that year. Only one positive DNA sample was successfully amplified by ITS1 nPCR. The RFLP analysis of the resulting 358 bp fragment showed a typical pattern of *L. infantum*, and the ITS1 partial sequence (GenBank: KY646445) was compared to those in GenBank, which confirmed the identity as *L. infantum* and *L. donovani* complex sequences (100% match). These results represent the first record of both *Leishmania* and *L. infantum* DNA from sand flies in Vojvodina and in Serbia.

Due to the low number of specimens sampled, we were not able to statistically compare different sampling techniques. However, most of the specimens were sampled by CDC (*n* = 80), then CDC/CO_2_ (*n* = 31) and the least by only CO_2_ (*n* = 25). No sand flies were sampled by sticky traps and mouth aspirators.

## Discussion

Sand fly research in Vojvodina was neglected for more than 60 years. Previous investigations conducted in the period 1949–1951 indicated a low diversity of sand flies, with only three species reported (*P. papatasi*, *P. perfiliewi* and *P. neglectus*) [18]*.* According to the old data *P. papatasi* was predominant species, being found in the majority of investigated locations in low numbers (1–2 specimens from various locations of the same village), while *P. neglectus* [19] and *P. perfiliewi* [20] were present in both, a limited number of locations and in the number of individuals (1–2 specimens from various villages).

Our results coincide partially with these data, confirming the presence of three previously reported species and indicating that, even in low numbers, *P. papatasi* remained the predominant species in Vojvodina, being found in 55% of all surveyed locations (91.67% of positive locations) (Table 1). Low densities of *P. perfiliewi* and *P. neglectus* were also confirmed; three specimens of *P. perfiliewi* were found (1 in 2013, 2 in 2015) and just one specimen of *P. neglectus* (2015).

The two specimens of *P. mascittii* represent first record for Vojvodina and the whole of Serbia, and may indicate a change in the composition of sand fly fauna in the region. *Phlebotomus mascittii* is a species found in Mediterranean region [21], as well as in countries with colder climate like Austria [22], Belgium [23] and Germany [24] with a northernmost border in Slovakia [25]. Considering its usually low abundance [26], and fact that species is present in countries bordering with our research area (e.g. Croatia [27] and Hungary [28]), there is a possibility that *P. mascittii* was already present in Serbia for several years, but remained undetected before 2015.

Patchy distribution and low density of sand flies is more prominent in Vojvodina than in other parts of Serbia, since Vojvodina is predominantly an agricultural region with a heavy use of insecticides. Large cultivated fields greatly influence sand fly dispersal potential and limit their distribution to small isolated clusters. Favourable weather condition, suitable topography and plentiful of food sources enable sand flies to maintain small, but steady, populations. Our data show that fragmented populations of sand flies are present across all of Vojvodina (Fig. 1).

Considering the increased number of canine leishmaniasis in Vojvodina in the past decade, and the presence of phlebotomine species competent vectors of *L. infantum*, all collected females were tested for the presence of *Leishmania*. Of 80 samples examined, three *P. papatasi* specimens resulted positive (4%) for *Leishmania* genus by nPCR. All positive females were collected in 2013. Negative results in 2014 and 2015 might be consequence of low number of samples (per locality and year), and low abundance of the vector species in general.

Although *P. papatasi* is a highly specific vector of *L. major* [29], presence of *L. infantum* DNA in *P. papatasi* has been previously reported [30]. Detection of leishmanial DNA does not imply that the sand fly species is a vector, as the assay cannot distinguish among developmental phases of promastigotes in the gut of infected specimens. According to Adler & Theodor [31], *P. papatasi* became infected after feeding on dogs with canine leishmaniasis, but the infection rate in the insects diminished continuously from 96% after one day, to 4% after 7 days (i.e. when the blood was digested). Our finding is most likely the direct result of recent blood-feeding, since all positive females had an abdomen full of blood, most likely engorged during the previous night(s).

Even though *L. infantum* DNA was detected from a non-competent vector, the fact that the parasite was identified from wild specimens, and the record of *Phlebotomus* (*Larroussius*) species proven vectors of *L. infantum* (*P. perfiliewi* and *P. neglectus*) [32, 33], indicates the possibility of *L. infantum* circulation in the surveyed region.

We wish also to highlight the importance of the *P. mascittii* record, since this species has long been suspected as a vector of *L. infantum* [34]. So far, *P. mascittii* has only been assumed to be a putative vector of *Leishmania* spp., however the recent detection of *L. infantum* DNA in specimens of *P. mascittii* from Austria [22] and Italy [21] may support possible competence in transmission (with the limitations exposed above about molecular assays for vector incrimination).

Despite the relatively low number of collected specimens, implication of leishmaniasis circulation in Vojvodina, and Serbia as a whole, seems to be accumulating. New cases of human autochthonous VL were recently registered in both past-endemic [35] and non-endemic areas of south-east Serbia [36]. Along with human and canine leishmaniasis cases, the presence of *Leishmania* spp. was also detected in the spleen of golden jackals (Mammalia, Canidae, *Canis aureus*) in central and east Serbia [37]. The possibility of vertical transmission among canine populations was discussed by Boggiatto et al. [38], and it is not excluded as a probable way of sustaining infection within wild and domestic canid population in Vojvodina/Serbia, since low numbers of sand flies were recorded. Beyond Serbia, reports of the disease are accumulating from all neighbouring countries [39–41]. Constant flow of humans, animals and commodities trough Vojvodina increases the risk of parasite introduction and disease emergence, since this region is situated in the main transit route of tourism and trade between the Mediterranean and Middle-eastern countries and Central and northern Europe.

## Conclusions

Presence of autochthonous canine leishmaniasis cases, detection of *L. infantum* DNA from wild-caught (non-competent) vector, and the record of *Phlebotomus* (*Larroussius*) species, which are proven vectors of *L. infantum* (*P. perfiliewi* and *P. neglectus*), indicate the possible dynamics of endemic circulation of *L. infantum* in the surveyed region.

## Abbreviations

CDC: Centre for Disease Control
CL: Cutaneous leishmaniasis
*cox*1: Cytochrome *c* oxidase subunit 1
ITS: Internal transcribed spacer
NGS: Next-generation sequencing
nPCR: nested PCR
RFLP: Restriction Fragment Length Polymorphism
SSU: Small subunit
VL: Visceral leishmaniasis
